# Long distance invisibility system to hide dynamic objects with high selectivity

**DOI:** 10.1038/s41598-017-10658-7

**Published:** 2017-08-31

**Authors:** Qiluan Cheng, Zuojun Tan, Hui Wang, Guo Ping Wang

**Affiliations:** 1 College of Science, Huazhong Agricultural University, Wuhan, 430070 Hubei P.R. China; 2 College of Electronic Science and Technology, Shenzhen University, Shenzhen, 518060 Guangdong P.R. China; 3 Institute of Information Optics, Zhejiang Normal University, Jinhua, 321000 Zhejiang P.R. China

## Abstract

With the development of invisibility technology, invisibility devices have now become more practical, with properties such as working at visible wavelengths, using natural materials, and hiding macroscopic objects. Recently, the cloaking of dynamic objects was experimentally realized using ray-optics. Here, based on a phase retrieval algorithm and phase conjugation technology, we design and fabricate a system to hide dynamic objects that changes at speeds faster than 8 seconds per frame. Different from shell cloaks and carpet-like cloaks, which conceal the entire region covered by the cloak, our system works when the object is at a distance and hides only the selected part of an object when the entire object is within the working area of the system. We experimentally demonstrate the concealment of a millimeter-scale object at different wavelengths. We believe that our work may provide a new approach to hiding objects in real life and may also be applicable in biological imaging and atmospheric imaging.

## Introduction

With the development of micro- and nanofabrication techniques, it has become possible to manipulate electromagnetic waves by modifying material parameters^1^, which presents opportunities to realize invisibility. Based on transformation optics^2^ and conformal mapping^3^, different invisibility devices, such as shell cloaks^4, 5^, carpet-like cloaks^6, 7^ and complementary medium-based cloaks^8, 9^, have been theoretically proposed and experimentally realized. Magnetic^10, 11^, acoustic^12, 13^ and thermal heat^14, 15^ invisibility devices have been designed and fabricated. In recent years, the manufacturing techniques and working conditions of invisibility devices have become increasingly practical to meet the requirements of hiding objects in real life, i.e., by working at visible wavelengths, using natural materials and hiding macroscopic objects^16, 17^. Furthermore, invisibility devices based on ray-optics have experimentally realized concealing dynamic objects^18–20^, even creatures, by causing light to propagate though a predefined path. Theoretically, shell cloaks^2–5, 18, 19^ and carpet-like cloaks^6, 7, 16, 17^ can hide dynamic objects. However, the object to be hidden must be enclosed or covered by the cloak, which not only makes the object invisible to the outside viewers, but also makes the outside invisible to the cloaked region. In addition, the entire region covered by the cloak is concealed without selectivity. Although complementary medium-based cloaks can hide objects outside of the cloaks, they can only hide static objects because the cloaks should be manufactured based on the object to be hidden, meaning that the cloaks themselves cannot change with the target object.

Here, we experimentally demonstrate a type of invisibility system that directionally hides dynamic objects which change over time. By combining a phase retrieval iterative algorithm with phase conjugation technique, we cause the system to produce a phase conjugated signal that changes with an object to compensate for the scattering light field of the object. Approximately 8 seconds pass between detection of the object and concealment of the object. In contrast to shell cloaks and carpet-like cloaks, the object hidden by our system is not covered by any optical devices. This system has high selectivity, meaning that it only conceals designated parts of an object while having no effect on other parts when the entire object is in the working area of the system. When the illumination light changes from one frequency to another or has a mixture of different frequencies, the hiding effect of our system is unchanged. This system works well when the dynamic object is at the millimeter scale. We believe that our system provides a practical way to hide dynamic objects selectively. Moreover, this system can also be extended to biological imaging and atmospheric imaging.

## Results

### Theoretical basis and experimental scheme

Phase conjugated light is widely used for imaging through a scattering medium^21–23^ and focusing beyond a diffraction limit^24, 25^. Physically, a phase conjugated signal can be used to compensate for the scattered light fields of an object, allowing it to be concealed^26^. In our previous work, we used traditional holography to create phase conjugation signals based on the time-reversal principle and experimentally fabricated dielectric devices to realize invisibility, create illusions^27–29^, and even image through a scattering medium^30^.

**Figure 1:**
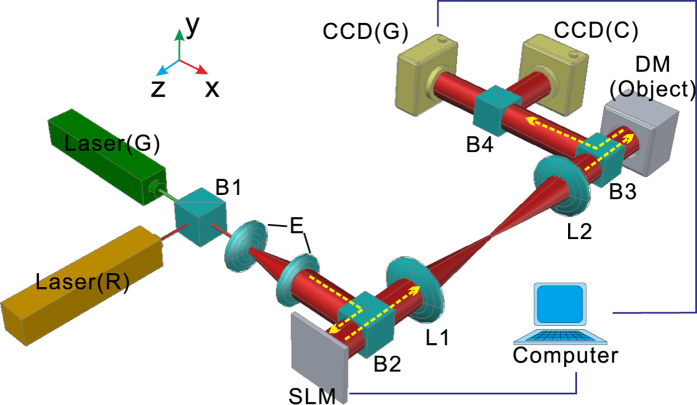
Experimental setup for hiding a dynamic object. A He-Ne laser beam with a wavelength of λ = 632.8 nm is reflected by split prism B1 and is collinear with a semiconductor laser beam with a wavelength of λ = 532.8 nm. After being expanded by beam expander E, the laser beam is reflected by split prism B2 and modulated by an SLM. The 4f system consists of two Fourier transform lenses L1 and L2 with a focal length f = 30 cm. The SLM and the deformable mirror DM are located at the front and rear focal planes, respectively, such that the SLM creates a one-to-one image on the DM plane. The object is generated by the DM. After being reflected by DM and split prism B3, the light beam is divided by split prism B4 into two parts: one part is received by a monochromatic charge-coupled device [CCD(G)] (Point GRAY), and the other is received by a color CCD [CCD(C)]. A feedback system is established between CCD(G) and SLM to control the SLM based on the photographs acquired by CCD(G). The hiding effect of the system is observed through CCD(C). The distance between DM and CCD(G) is 302 mm, and the distance between CCD(C) and DM is 400 mm.

Following our previous work, we combine a phase retrieval iterative algorithm^31, 32^ and phase conjugation technique in this paper to create a phase conjugated signal that changes with a dynamic object to hide it. The invisibility system is illustrated in Fig. 1. A deformable mirror (DM) (Thorlabs, A0120INF-03, America), which is used to generate a dynamic phase-modulated object, and a spatial light modulator (SLM) [Boulder Nonlinear Systems (BNS), FP512, America], which is used to modulate the wavefront of the light source and create phase conjugated signals to hide the object, are placed at the rear and front focal planes, respectively, of a 4f optical system consisting of lenses L1 and L2. Thus, the pattern displayed on the SLM is one-to-one imaged on the object plane. To obtain the object information, we input five different known random phase distribution maps into the SLM and obtain five photographs from a monochromatic charge-coupled device [CCD(G)]. By using the phase retrieval iterative algorithm^33^, the computer deduces the entire complex light field on the object plane. Based on the calculation results, the computer commands the SLM to produce light with phase conjugated signals to hide selected parts of the object. By using a feedback system composed of the CCD(G), a computer, and the SLM, we cause the phase pattern displayed on the SLM to change with the object to dynamically hide it. A color CCD [CCD(C)] is used to observe the working effect of our system.

### Hiding a dynamic object under monochromatic light illumination

**Figure 2:**
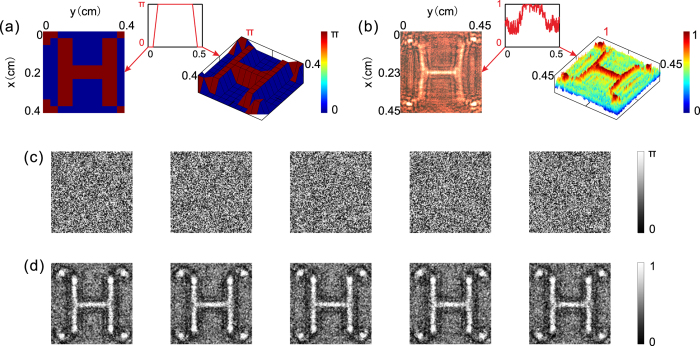
Signals input and detected in the experiment. (**a**) Planar image of the phase distribution input into the DM and its 3D profile (right-hand panel). The inset line graph shows the phase distribution along the y-axis at x = 2 mm. (**b**) Photograph (left-hand panel) received by CCD(C) and its 3D intensity profile (right-hand panel) when the object displayed on DM is directly illuminated by planar light. The inset of (**b**) shows a line graph of the corresponding image along the y-axis at x = 2.3 mm. (**c**) 5 phase distributions input into SLM during the process of retrieving the object phase. (**d**) 5 photographs received by CCD(G) corresponding to different phase distributions of SLM.

As a dynamic object, the deformable mirror displays 4 different letters (H, Z, A, and U) in turn. Each letter is surrounded by four points, and each image is displayed for 8 seconds. We take the first frame of the object as an example to explain the working effect of our system. The phase distribution, which we input into the deformable mirror as the original object, is shown in Fig. 2a. To provide a quantitative description, we show a three-dimensional (3D) profile (right panel) in Fig. 2a and an inset line graph, which depicts the phase distribution of the object along the y-axis at x = 2 mm. The phase difference between the letter H with four points and the background is set as π. Note that the original object phase distribution is unknown throughout the entire process of hiding the object. We provide Fig. 2a to contrast with the retrieved object phase distribution.

When the object is directly illuminated by an expanded laser beam at λ = 632.8 nm, we can see the image of H surrounded by four points in the photograph taken by CCD(C) (Fig. 2b), demonstrating that the entire object is visible without our system. Although the object is phase-modulated, the letter and four points are visible because of the diffraction effect. To quantitatively determine the contrast of the images, we show a 3D profile in the right panel and an intensity line graph of the image along the y-axis at x = 2.3 mm in the inset. The bulge in the line graph corresponds to the horizontal stroke in the middle of the letter H. To obtain the object information, we input 5 different random phase distribution maps (Fig. 2c) in turn into the SLM. The phase fluctuation is known and is set between 0 and π. In this situation, CCD(G) takes 5 black-and-white photographs, in which the shade of gray represents the intensity of the light field (Fig. 2d).

**Figure 3:**
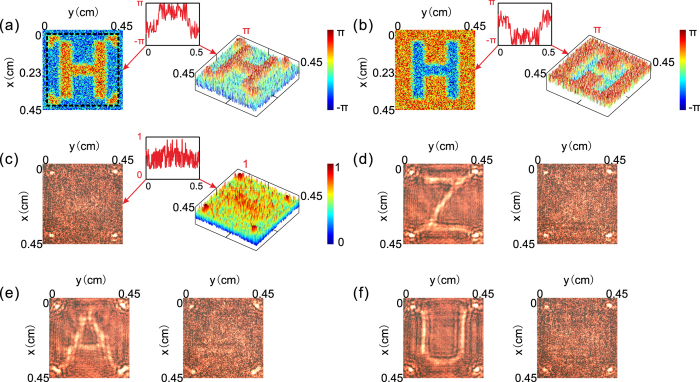
Experimental results of hiding a dynamic object. (**a**) Planar image (left-hand panel) of the retrieved object phase distribution and its 3D intensity profile (right-hand panel). (**b**) Planar image (left-hand panel) and a 3D profile (right-hand panel) of the phase distribution input into SLM to produce the phase conjugated signal. (**c**) Photograph (left-hand panel) received by CCD(C) and its corresponding 3D intensity profile (right-hand panel) when the phase distribution in Fig. 3(b) is input into SLM. (**d**–**f**) Photographs taken by CCD(C) when our system hides different frames of the dynamic object. The left-hand panel shows the photograph taken when the object is directly illuminated by planar light, while the right-hand panel shows the photograph taken when the object is hidden by our system. The insets of (**a**–**c**) show line graphs of the corresponding images along the y-axis at x = 2.3 mm.

Using the phase retrieval iterative algorithm (the concrete steps of the algorithm are described in the Methods section), the computer recovers the entire complex light field on the object plane based on the 5 photographs shown in Fig. 2d. The retrieved object phase distribution is shown in Fig. 3a, from which we can clearly distinguish the pattern of the letter H and its four points. To illustrate the feature properties of the phase distribution, we also present a 3D profile on the right in Fig. 3a and a line graph of the phase distribution along the y-axis at x = 2.3 mm in the inset. Ignoring the noise, we see that the phase difference between the areas of H with four points and other areas is π, meaning that the retrieved phase distribution agrees with the original object phase distribution. The specific reasons for the differences between Figs 3a and 2a are detailed in the discussion section.

To hide the letter H without changing the four surrounding points, the computer identifies the signal of the letter and determines its conjugated phase distribution. Based on the calculation result, the computer inputs the proper phase distribution (Fig. 3b) into the SLM. In this case, the photograph taken by CCD(C) (Fig. 3c) only contains the image of four points. To quantitatively illustrate the experimental result, we show its 3D profile in Fig. 3c, and the intensity distribution of the image along the y-axis at x = 2.3 mm in the inset. Unlike Fig. 2b, there are no signals of the letter H, while the image of the four surrounding points is still visible, demonstrating that our system conceals the letter H without affecting the four points that are not selected.

When CCD(G) detects changes in the object, the invisibility system repeats the process of obtaining the object information and producing a phase conjugated signal to hide the new letter without altering the four points. In the experiment, each random phase distribution map is set to stay for 0.3 seconds on the SLM to give the CCD(G) enough time to record the intensity distribution and transmit the images to the computer. It takes the computer approximately 5 seconds to retrieve the object information and input an appropriate phase distribution map into the SLM to hide the letter. Thus, we set each frame of the object to remain for 8 seconds to ensure that our system has sufficient time to complete the hiding process before the object changes. A movie illustrating the entire process of hiding the dynamic object is provided in the supporting online materials (Supplementary Movie S1). In the movie, it is clear that the letter is invisible at the end of each frame, while the four surrounding points are unchanged. This result demonstrates that if an object changes at a speed of 8 seconds per frame, our system can hide each frame before it changes with high selectivity.

Figure 3d–f show the hiding effect of each frame of the object. When illuminated by planar light, we can see images of the four points of letters Z, A and U, respectively, from the photographs received by CCD(C) (left panels of Fig. 3d–f). With our system, the photographs contain only the image of the four points without any information of the letters (right panels of Fig. 3d–f), indicating that our system conceals the letter, which is identified from each frame, without changing the four points around it.

### Working effect of the system under different conditions

**Figure 4:**
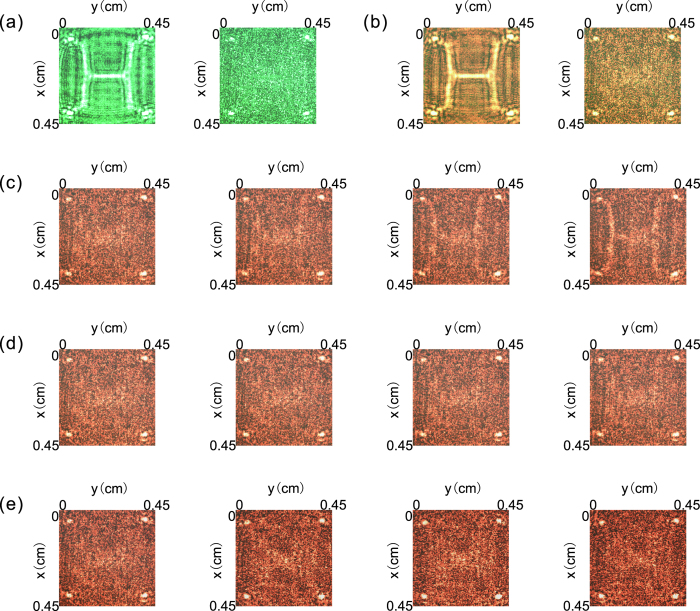
Hiding effect of our system under different conditions. (**a**) The working effect of our system when the illumination light is green. (**b**) The hiding effect of our system when the illumination light is a mixture of red and green. The left panels of (**a**,**b**) show photographs taken by CCD(C) when the object is directly illuminated by planar light; the right panels are taken when the object is hidden by our system. (**c**) Photographs received by CCD(C) when the SLM moves 45 μm, 90 μm, 135 μm, and 180 μm from its original position along the x-axis. (**d**) Photographs received by CCD(C) when the SLM moves −200 μm, −100 μm, 100 μm, and 200 μm from its original position along the z-axis. (**e**) Photographs received by CCD(C) when the orientation deviation of DM is 5°, 10°, 15°, and 20° from its original orientation around the y-axis.

Because that both the DM and the SLM can work across the visible wavelength range, we change the illumination light from red laser light at λ = 632.8 nm to green laser light at λ = 532 nm (the green light comes from a semiconductor laser) to verify the working effect of our system under different illumination conditions. We also take the first frame of the dynamic object as an example to explain the results. When the illumination light changes to green, the photograph received by CCD(C) shows the image of both the letter H and its four points (left panel of Fig. 4a) without our system, while a photograph containing only the image of the four points is obtained when our system is working (right panel of Fig. 4a). Moreover, because the illumination light in practical situations is always non-monochromatic, we turned on both Laser(R) and Laser(G), as shown in Fig. 1, to create an illumination light mixture of different laser beams. The photographs taken by CCD(C) with (right panel of Fig. 4b) and without (left panel of Fig. 4b) our system indicate that our system works well when illuminated by light with multiple wavelengths.

A movie illustrating the working effect of our system when the illumination light changes is provided in the supporting online materials (Supplementary Movie S2). As shown, if the object is successfully concealed by our system under the illumination of one monochromatic light, the hiding effect remains unchanged when the illumination light changes.

Theoretically, if the SLM does not perfectly perform one-to-one imaging on the object plane, the hiding effect of our system may be seriously inhibited. However, the image of the SLM may not entirely coincide with the object displayed on the deformable mirror in the experiment. Thus, we verify the effect of position and orientation deviations of the SLM and DM when hiding objects. The four photographs in Fig. 4c are taken by CCD(C) when the SLM is transversally deviated 45 μm, 90 μm, 135 μm, and 180 μm from its original position along the x-axis, from left to right. As shown, when the transversal deviation becomes larger than 135 μm, the letter H is visible. Figure 4d exhibits the photographs acquired by CCD(C) as the SLM deviates longitudinally by −200 μm, −100 μm, 100 μm, and 200 μm from its original position along the z-axis, from left to right. The pictures contain only the information of the four points, meaning that such large longitudinal deviation is still tolerable for hiding the object. In Fig. 4e, we show photographs taken by CCD(C) when the orientation deviation of the deformable mirror is 5°, 10°, 15°, and 20° from its original orientation around the y-axis, from left to right. The letter H is hidden, while the four points are visible in these photographs, demonstrating that our system is not sensitive to rotation of the object. All experimental results in Fig. 4c–e are numerically simulated in Fig. S1 in the Supplementary Information. The above experimental results indicate that the system still works well when the SLM image does not perfectly coincide with that of the deformable mirror. This result occurs because the background noise conceals parts of the image.

## Discussion

Compared with Fig. 2a, the outline of the retrieved phase distribution in Fig. 3a is smoother and more blurred. This phenomenon occurs because in the experiment, we use a continuous surface deformable mirror with a discrete drive. The surface of the deformable mirror experiences no sudden change, while the gradient at the edge between areas corresponding to different input values does; that is, although we input a pattern with sharp edges, the pattern displayed on the DM has a smooth contour. The outline of the retrieved phase distribution is different from the pattern we input into the DM but is similar to the pattern that is actually displayed on the DM. Thus, the outline of the pattern in Fig. 3a is smoother than that in Fig. 2a.

Except for the errors introduced in the experimental operation and calculated approximation, the noise points in Fig. 3a are primarily due to differences between the theoretical conditions used in the phase retrieval iterative algorithm and actual experimental conditions. To simplify the calculation in the feedback system, we assume that the optical path length between the object and CCD(G) is 320 mm in the phase retrieval iterative algorithm. The calculation process for the theoretical optical path length is detailed in the Methods section. However, in the experiment, the actual optical path length from DM to CCD(G) differs from the theoretical value. As shown in the simulation results in Fig. S2 in the Supplementary Information, if the difference is less than 5 mm, then the working effect of our system is unaltered. Moreover, the 5 photographs in Fig. 2d lose the highest and lowest parts of the light intensity because of the threshold of CCD(G). This effect also causes the retrieved object phase pattern in Fig. 3a to have noise points. The simulation results of the negative influences caused by the threshold of CCD(G) are shown in Fig. S3.

In our experiment, a duration of 8 seconds is required to completely hide one frame of the object, which limits the changing speed of the dynamic object. As the response time of the SLM is less than 30 ms, this limitation is largely due to the computing power of the computer used in the feedback system. If the time between detecting an object and hiding it is less than the eye response time, the system can hide a dynamic object from the eye in real time. Moreover, this system can be extended to imaging through a scattering medium by selectively hiding the scattering layer from the target object. If the time required for the hiding process is optimized to the millisecond scale, our system can be used in biological imaging by removing the scattering effect of the dynamic biological tissue layer covering the target object. If the change rule of the dynamic object is known, we can calculate the pattern that is displayed on the SLM in advance. When the object appears, the SLM can directly produce light containing phase conjugation signals to hide the object in real time without the process of retrieving the object information.

To get a better result, our system is limited to hide an object which has reflective surface in the experiments. Nevertheless, our system can be used to hide objects which shows scattering strong enough, such as some scattering tissues in the biological imaging, in principle. In addition, by using the same principle, the system can be extended to hide thin transmission phase-modulated objects. Because of the phase retrieval iterative algorithm and the feedback system which we used in the experiments, our system can only work for coherent light sources. Coherent light source, including lasers, is also applicable in some fields, such as atmospheric imaging. For example, our system can be extended to selectively hide atmosphere without changing the information of the object to be detected. Then one can remove the influence of the atmosphere in the imaging process. Thus, despite of those limitations, our system is practical in fields such as biological concealing and atmospheric imaging.

In conclusion, we have reported a type of invisibility system to directionally conceal dynamic objects. By combining a phase retrieval iterative algorithm with phase conjugation techniques, we experimentally establish a system that contains a feedback loop consisting of a monochromatic CCD(G) to detect object information and an SLM to produce a phase conjugated signal that changes with the object. The time between detecting the object and creating the phase conjugated signal is approximately 6.5 seconds, which allows our system to be applied to dynamic objects with a changing speed of 8 seconds per frame. Furthermore, our system is highly selective; it hides only the selected portions of the object without affecting the other parts when the entire object is covered by the modulated light created by our system. The system works for macroscopic objects (at the millimeter scale) at visible wavelengths when the object is not enclosed or covered by any optical devices. Moreover, the system can operate when the wavelength of the illumination light changes, even when the light source has multiple wavelengths. We believe that our work may provide new ideas for hiding real objects and may also be applicable in biological imaging and atmospheric imaging.

## Methods

We assume that the five known random phase distributions displayed on the SLM in turn are ${\phi }_{m}({{\rm{x}}}_{0},{{\rm{y}}}_{0})$ (m = 1, 2, …, 5) and that the amplitude distributions received by CCD(G) at the detection plane are A_m_(x_1_,y_1_), corresponding to each ${\phi }_{m}({{\rm{x}}}_{0},{{\rm{y}}}_{0})$.

1) Randomly choose an initial estimation of ${\theta }_{m}({{\rm{x}}}_{1},{{\rm{y}}}_{1})$ and combine it with the detected amplitude ${A}_{m}({{\rm{x}}}_{1},{{\rm{y}}}_{1})$ to construct ${U}_{m}({{\rm{x}}}_{1},{{\rm{y}}}_{1})={A}_{m}({{\rm{x}}}_{1},{{\rm{y}}}_{1}){\rm{e}}{\rm{x}}{\rm{p}}[-{\rm{i}}{\theta }_{m}({{\rm{x}}}_{1},{{\rm{y}}}_{1})]$ as the complex light field on the detection plane.

2) Propagate ${U}_{m}({{\rm{x}}}_{1},{{\rm{y}}}_{1})$ back to the object plane using the Fresnel algorithm and obtain an estimated complex light field $V{\text{'}}_{m}({{\rm{x}}}_{0},{{\rm{y}}}_{0})$.

3) Set the complex amplitude of the light field on the object plane as ${V}_{m}({{\rm{x}}}_{0},{{\rm{y}}}_{0})={V}_{m}^{^{\prime} }({{\rm{x}}}_{0},{{\rm{y}}}_{0})/\exp [-{\rm{i}}{\phi }_{m}({{\rm{x}}}_{0},{{\rm{y}}}_{0})]\,\times$ $\exp [-{\rm{i}}{\phi }_{m+1}({{\rm{x}}}_{0},{{\rm{y}}}_{0})]$. Propagate ${V}_{m}({{\rm{x}}}_{0},{{\rm{y}}}_{0})$ to the detection plane using the Fresnel algorithm and obtain a new complex light field ${U}_{m+1}^{^{\prime} }({{\rm{x}}}_{1},{{\rm{y}}}_{1})={B}_{m+1}({{\rm{x}}}_{1},{{\rm{y}}}_{1})\exp [-i{\theta }_{m+1}({{\rm{x}}}_{1},{{\rm{y}}}_{1})]$.

4) Replace ${B}_{m+1}({{\rm{x}}}_{1},{{\rm{y}}}_{1})$ with the detection amplitude ${A}_{m+1}({{\rm{x}}}_{1},{{\rm{y}}}_{1})$ in *U*′_*m*+1_(x_1_, y_1_) and combine a new complex light field ${U}_{m+1}({{\rm{x}}}_{1},{{\rm{y}}}_{1})={A}_{m+1}({{\rm{x}}}_{1},{{\rm{y}}}_{1})\exp [-{\rm{i}}{\theta }_{m+1}({{\rm{x}}}_{1},{{\rm{y}}}_{1})]$. Set m = m + 1 and use ${U}_{m+1}({{\rm{x}}}_{1},{{\rm{y}}}_{1})$ as the new object complex light field ${U}_{m}({{\rm{x}}}_{1},{{\rm{y}}}_{1})$ in step 2.

5) Repeat steps 2–4 until m = 5. Replace m + 1 with 1 in the last loop. Obtain ${W}_{n}({{\rm{x}}}_{0},{{\rm{y}}}_{0})={V}_{5}^{^{\prime} }({{\rm{x}}}_{0},{{\rm{y}}}_{0})/$ $\exp [-{\rm{i}}{\phi }_{5}({{\rm{x}}}_{0},{{\rm{y}}}_{0})]$ as the estimated complex light field on the object plane.

6) Repeat steps 2–5 until the difference between two successive retrieved wave fronts ${W}_{n}({{\rm{x}}}_{0},{{\rm{y}}}_{0})$ and ${W}_{n-1}({{\rm{x}}}_{0},{{\rm{y}}}_{0})$ (n is the number of iterations throughout the loop from 2 to 5) is sufficiently small. The complex angle of ${W}_{n}({{\rm{x}}}_{0},{{\rm{y}}}_{0})$ is the calculated object phase distribution.

The phase retrieval iterative algorithm described above is based upon the Fresnel approximation algorithm. To reduce the iteration computation, the number of pixels of the SLM, DM and CCD(G), and the distance between the object and CCD(G) are carefully designed. In the calculation, we set the size of the SLM, CCD(G), and the object as 4.5 × 4.5 mm and the number of pixels as 100 × 100. According to the sampling interval relationship in the Fresnel algorithm, the distance between DM and CCD(G) is $d={l}^{2}/N/\lambda$, where *l* is the side length of the object, *N* is the number of pixels on one side of the object, and *λ* is the wavelength of the illumination light. Thus, we can determine that CCD(G) should be placed 320 mm from the deformable mirror. To avoid the influence of the optical prism, the real distance between CCD(G) and DM is 302 mm.

In the experiment, the pixel number of CCD(G), SLM and DM can be adjusted to 100 × 100 via interpolation. Because the receiving area of CCD(G) and the display area of the SLM are both larger than 4.5 × 4.5 mm, we can use part of these areas to meet the calculation conditions of the phase retrieval iterative algorithm. However, the deformable mirror, with a display area of 4 × 4 mm, does not match the assumed size. In practice, we can treat the areas around the display screen of the DM as part of the object because these areas reflect light as a plane mirror. Thus, the reconstructed object information corresponds to a larger area that is covered by the image of the SLM on the DM. In the retrieved object phase distribution map shown in Fig. 3a, the middle part, which is surrounded by a dashed frame, corresponds to the area of the original pattern that we input into the DM.

## Electronic supplementary material

Supplementary Information

Movie 1

Movie 2
