# Supplementary material for: Long distance invisibility system to hide dynamic objects with high selectivity
Source: Sci Rep. 2017 Aug 31;7:10231. doi: 10.1038/s41598-017-10658-7 (PMC5579039; doi:10.1038/s41598-017-10658-7)
Supplement: Supplementary file 1 — Supplementary Information [file 41598_2017_10658_MOESM1_ESM.pdf]

SUPPLEMENTARY INFORMATION

Long distance invisibility system to hide dynamic objects with high selectivity

Authors: Qiluan Cheng[1], Zuojun Tan[1], Hui Wang[3], and Guo Ping Wang[2*]

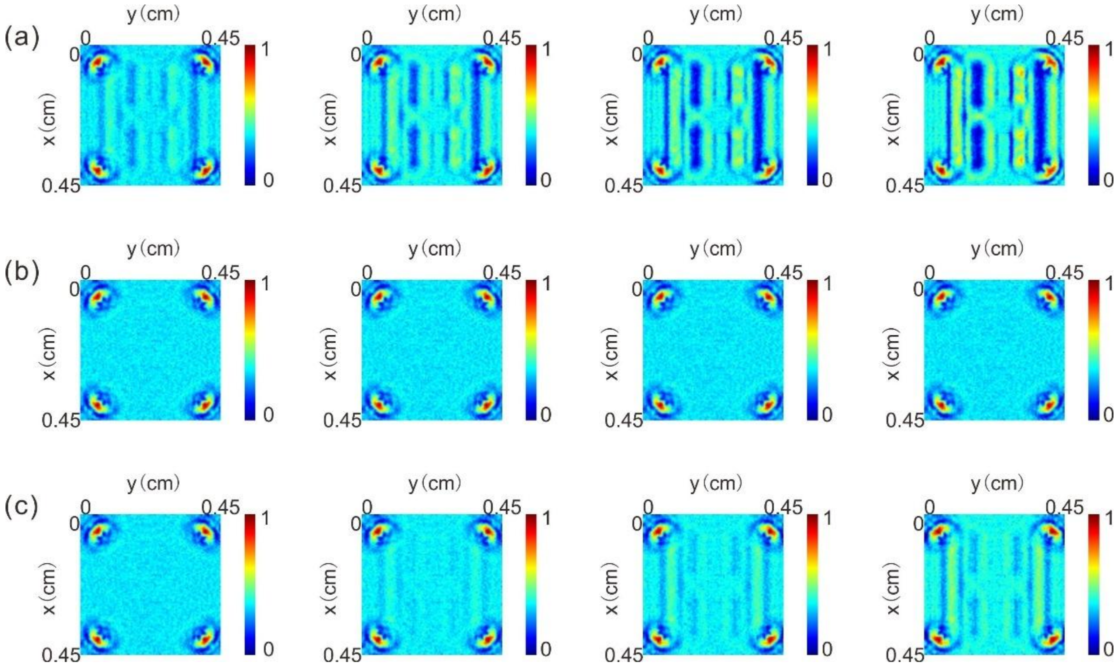

Figure S1: Simulation results of hiding the first frame of the object when the SLM or DM is moved from its original position. (a) Planar image of the intensity distribution on the observation plane [the plane of CCD(C)] when the SLM is transversally deviated 45 μm, 90 μm, 135 μm, and 180 μm from its original position along the x-axis. (b) Planar image of the intensity distribution on the observation plane when the SLM is longitudinally deviated -200 μm, -100 μm, 100 μm, and 200 μm from its original plane along the z-axis. (c) Planar image of the intensity distribution on the observation plane when the DM is rotated 5 °, 10 °, 15 °, and 20 °from its original orientation around the y-axis.

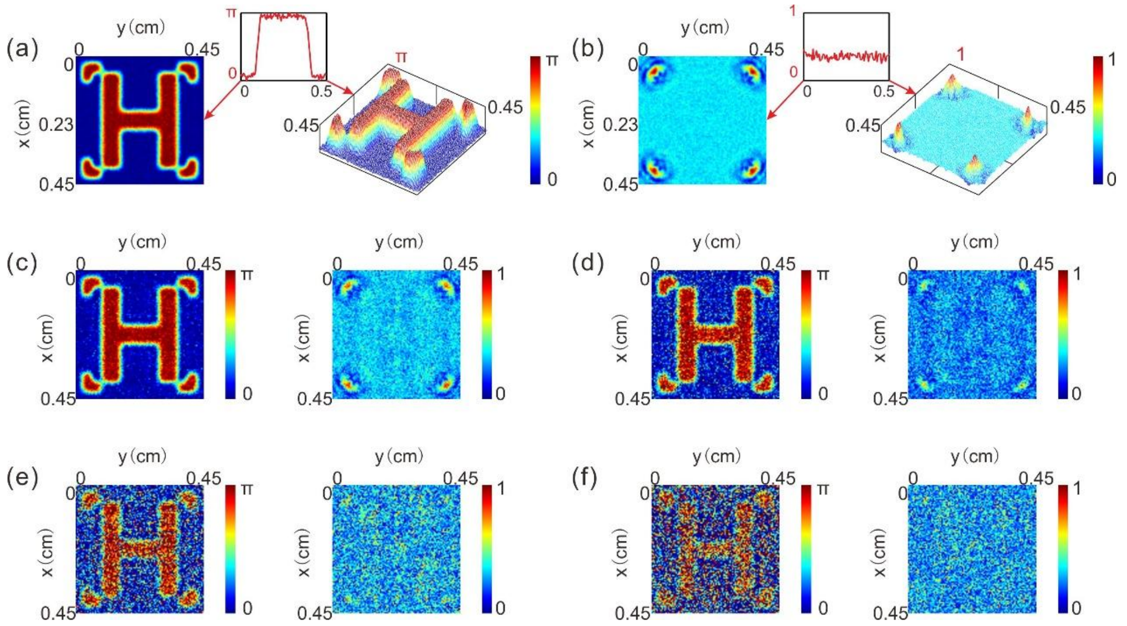

Figure S2: Simulation results of the working effect of our system when the real distance between the DM and CCD(G) in the experiment and the theoretical distance used in the phase retrieval algorithm differ. (a) Retrieved object phase distribution map when the real distance in the experiment and the theoretical distance are the same. (b) Intensity distribution map at the observation plane when the two distances are the same. (c-f) The retrieved object phase distributions (left) and the intensity distributions received at the observation plane (right) when the difference between the real distance and the theoretical distance is 3 mm, 5 mm, 7 mm, and 10 mm, respectively.

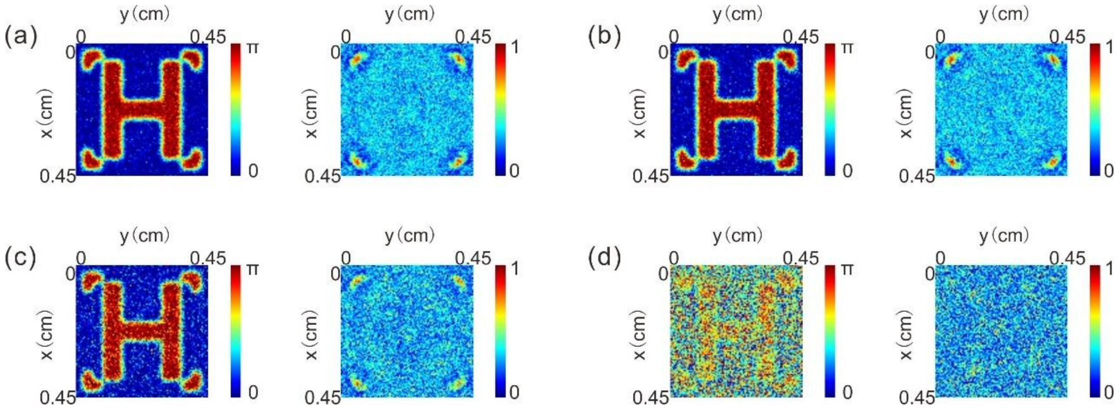

Figure S3: Simulation results of the working effect of our system when the CCD(G) loses the highest and lowest parts of the light intensity. (a-d) The retrieved object phase distributions (left) and the intensity distributions at the observation plane (right) when the intensity threshold of CCD(G) is 0-0.830, 0.67, 0.17-1, and 0.33-1, respectively. In the simulation, we assume that the entire intensity range at the detection plane of CCD(G) is 0-1.

By comparing the planar images in Figs. S2(a)-(b) with those in Figs. S2(c)-(f), we determine that if the distance between DM and CCD(G) in the experiment and that in the phase retrieval iterated algorithm are different, the reconstruction object phase patterns have noise points. The difference further leads to noise points in the intensity pattern on the observation plane. As the difference increases, the noise becomes serious. If the difference is less than 5 mm, our system can still hide the object. Otherwise, the noise points cover all of the signals on the observation plane, including the images of the four points, which are not chosen to be hidden.

In comparing Fig. S3 with Figs. S2(a)-(b), we determine that the intensity threshold of CCD(G) influences the retrieving effect of the object phase distribution and causes noise points in the intensity patterns received by CCD(C). In the right panel of Figs. S3(a) and S3(b), the noise caused

by the intensity threshold of CCD(G) does not influence the working effect of our system, meaning that losing the high part of the light intensity received by CCD(G) has a limited effect on hiding the letter. However, in the right panel of Figs. S3(c) and S3(d), the noise caused by the intensity threshold of CCD(G) is more serious. We fail to retrieve the object phase distribution when the intensity threshold of CCD(G) is 0.33-1 [left panel of Fig. S3(d)]. Thus, the low part of the intensity in the detection plane is very important in the process of retrieving the object phase. Based on these simulation results, we decrease the lowest threshold value of CCD(G) in the experiment to ensure that the low part of the light intensity on the detection plane is recorded by CCD(G).

Movie Legends

Supplementary Movie 1

This movie, taken by CCD(C), illustrates the process of hiding a dynamic object when the illumination light is at a wavelength of 632.8 nm. The words at the bottom of the frame represent different stages in the process of hiding the object; “Object” indicates that the object is directly illuminated by planar light, “Scatter” indicates that the SLM displays 5 different random phase distribution maps in turn, “Retrieval” implies that the computer is calculating the proper phase distribution, which is input into the SLM to produce the phase conjugated signal, and “Hidden” indicates that the object is hidden by our system (QuickTime; 1,023 KB).

Supplementary Movie 2

This movie, taken by CCD(C), illustrates the dynamic process of hiding an object when the illumination light changes. The illumination light changes from a monochromatic red laser beam at 632.8 nm to a multicolor laser beam with a mixture of red light at 632.8 nm and green light at 532 nm and then to a monochromatic green laser beam at 532 nm (QuickTime; 145 KB).
